# The aesthetic perception of orthodontic specialists, general dentists and laypeople regarding different smile displays for a patient missing one upper lateral incisor and the other one peg-shaped

**DOI:** 10.3389/fdmed.2025.1532220

**Published:** 2025-03-11

**Authors:** Federica Montinaro, Ludovica Nucci, Maria Chiara Chiarenza, Fabrizia d’Apuzzo, Letizia Perillo, Vincenzo Grassia

**Affiliations:** ^1^ Multidisciplinary Department of Medical-Surgical and Dental Specialties, University of Campania Luigi Vanvitelli, Naples, Italy; ^2^ Department of Mental and Physical Health and Preventive Medicine, University of Campania Luigi Vanvitelli, Naples, Italy

**Keywords:** smile aesthetic, missing incisor, peg-shaped tooth, aesthetic, missing lateral incisor

## Abstract

**Background:**

The prevalence of maxillary lateral incisor agenesis ranges from 1% to 3%, with slight global variability. The unilateral agenesis of the maxillary lateral incisor is often associated with a contralateral tooth with microdontia or a peg shape and can have esthetic, functional, and psychosocial implications for patients. The aim of the present survey was to assess the perceptions of smile aesthetics among orthodontists, general dentists and laypeople on different treatment choices, modifying the initial condition of the right maxillary lateral incisor agenesis and the contralateral peg-shaped tooth.

**Methods:**

A series of 6 photographs of different smile simulations were presented to 109 orthodontic specialists, 109 general dentists and 141 laypeople through an online survey. Each photograph was duplicated and judged from 1 to 10 for 2 different bipolar adjectives.

**Results:**

Statistically significant results were found for all the groups investigated. All three groups preferred the photo that maintained the typical symmetrical ‘high-low-high gingival contour. Moreover, both orthodontic specialists and general dentists preferred unilateral mesialization of the canine and conservative rehabilitation of the peg-shaped incisor. In contrast, the laypeople preferred bilateral mesialization of the canines with peg-shaped incisor avulsion.

**Conclusion:**

The normal symmetrical array of the central incisor, lateral incisor and canine had the best aesthetic results for all subjects. Laypeople were more attracted to a symmetrical smile than were the groups of orthodontic specialists and general dentists.

## Introduction

Excluding third molar, maxillary lateral incisor agenesis (MLIA) is the most common congenitally missing permanent tooth condition in the maxillary anterior region, accounting for nearly 20% of all missing teeth. The prevalence of maxillary lateral incisor agenesis reported in literature ranges from 1% to 3%, with slight global variability (1–3).

This condition occurred 1.4 times more often in females than in males, and it was more common bilaterally (4). There was a correlation between the congenital absence of teeth and the generalized reduction in the width of the teeth (5). Moreover, unilateral agenesis of the maxillary lateral incisor is often associated with a contralateral tooth with microdontia or a peg shape (6, 7). The absence of a maxillary lateral incisor with or without an anomalous contralateral incisor seriously compromises the aesthetics and harmony of the smile (8). The harmony and balance of the smile impact people's quality of life, increasing their self-esteem and interpersonal relationships. For this reason, the aesthetic need should not be underestimated, and it is one of the main objectives of the treatment choice (9, 10).

Several factors influence the smile aesthetics, as suggested by Machado (11) with the “Ten Commandments” for smile aesthetics, such as:

1. Smile Arc: Maxillary incisors should be positioned vertically.
2. Maxillary Central Incisors Ratio and Symmetry: Emphasis on proportionality and harmony.
3. Anterosuperior Teeth Ratio: Proper dimensional relationships among the anterior maxillary teeth.
4. Presence of Anterosuperior Space: Adequate spacing to ensure an aesthetically pleasing arrangement.
5. Gingival Design: Optimal shape and contour of the gingiva.
6. Levels of Gingival Exposure: Appropriate visibility of gingival tissue during a smile.
7. Buccal Corridor: Balanced lateral spaces between the buccal surfaces of posterior teeth and the lips.
8. Midline and Tooth Angulation: Alignment and proper angulation of the dental midline and teeth.
9. Details: Consideration of tooth color and anatomical shape for natural and appealing results.
10. Lip Volume: The lips should complement the overall smile harmony.

Ricketts explained how the harmony and balance of the smile were based on the golden proportions between every tooth, especially for the anterior sextant. The golden proportions were also evident between dentition and face (12). The literature has clarified many aspects of the perception of the attractiveness of the smile among laypeople, dentists and orthodontists. Magne et al. (13) showed how visual attention went first to the central incisor and then to the canine, paying less attention to the lateral incisor. Kokich et al. (14) reported that orthodontists, general dentists and laypeople had different perceptions of smile alterations with different levels of deviation. They showed that small dental aesthetic discrepancies were not even highlighted by most patients. Pini et al. (9) reported that golden proportions are not found in most patients treated for lateral incisor agenesis, whether unilateral or bilateral. According to many studies, a symmetrical smile has a more pleasing aesthetic, including symmetry in the height of the gingival margins (13, 15, 16). To treat maxillary lateral incisor agenesis, the options available were to close the space with orthodontics only (17–19) or to open the space and reposition the absent lateral incisor with prosthetic rehabilitation (bridge) or with a single implant-borne crown (20–23). The most appropriate therapeutic alternative depends on several factors, such as the morphology and color of the canine, the smile line, the presence or absence of malocclusion, the presence or absence of space in the agenesis area, the profile, the pattern of growth, etc (24, 25).

The literature did not establish whether one of the two treatments was better than the other because of the lack of RCTs, but both options had specific advantages and disadvantages that needed to be carefully considered by clinicians (26, 27).

Indeed, space closure through the mesialization of the posterior teeth allowed the treatment to conclude without implant-prosthetic rehabilitation. At the other site, this option requires a final crown reshaping of the canine and the first premolar to simulate crown morphology of the lateral incisor and the canine, respectively (17).

Alternatively, the space opening option, in which the canine is left in its natural position, requires prosthetic-implant rehabilitation that substitutes for the missing lateral incisor (20).

The aesthetic result of this treatment choice was highly dependent on the anatomical characteristics of each patient, the intraoperative procedures, and the collaboration between the different specialists.

In addition, the insertion of an implant requires strict control of periodontal support, which over time decreases the risk of compromised smile aesthetics (28).

Jamilian et al., evaluating both treatments with a 5-year follow-up, demonstrated that patients who had closed the space by mesialization of the canine with orthodontics had better periodontal health. In addition, all the patients with the implant presented an infraocclusion equal to or greater than 1 mm (29). Furthermore, agenesis of the upper lateral incisor is in some cases associated with the corresponding contralateral anomalous or peg-shaped incisor (5–7).

The treatment plan for this specific clinical condition was even more complex, with more treatment options available to ensure a functional and aesthetic. The rehabilitative evaluations of the peg-shaped tooth included increasing the space associated with reshaping by prosthetic or adhesive techniques or closing the space by orthodontics and peg-shaped tooth extraction.

There are a few case reports concerning the perception of smile aesthetics and the therapeutic options in patients with maxillary lateral incisor agenesis associated with contralateral peg-shaped incisor (30, 31). The smile aesthetics and post treatment perception of this initial clinical condition required assessments for the most appropriate treatment.

The aim of the present survey was to assess the perceptions of smile aesthetics by orthodontists, general dentists and laypeople regarding different treatment choices, modifying the initial condition of the right maxillary lateral incisor agenesis and the contralateral peg-shaped tooth.

The results of this survey could aid clinicians in the decision-making processes for different treatment options.

## Materials and methods

The study was approved by the ethical committee of the University of Campania, Luigi Vanvitelli, according to the Declaration of Helsinki.

**Figure 1:**
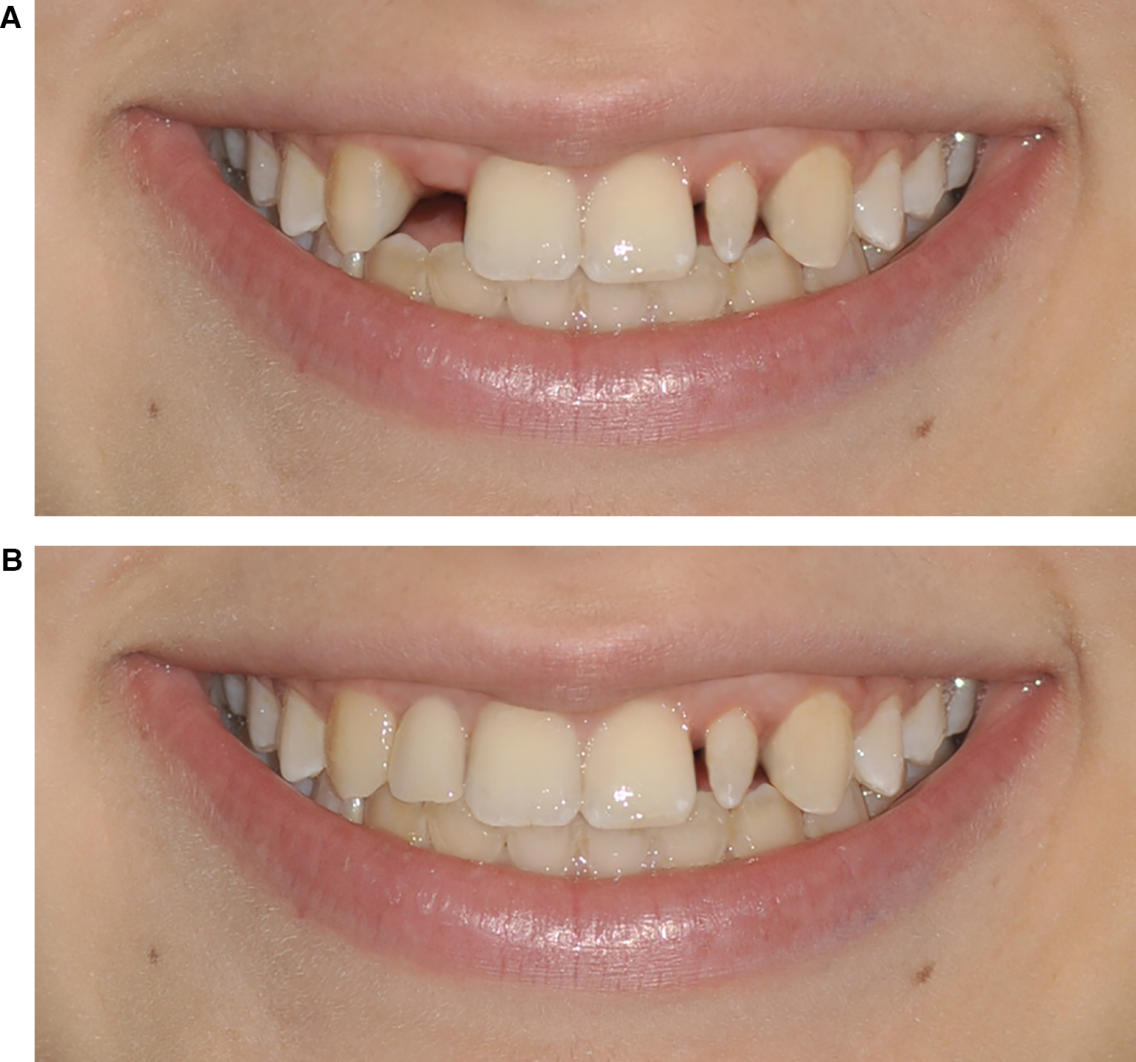
**(A)** Photographs n.3 and n.9. Patient's original smile with the maintenance of the space between the upper right lateral incisor and the upper left lateral peg-shaped incisor. **(B)** Photographs n.5 and n.7. The upper right lateral incisor with implant rehabilitation and maintenance of the upper left lateral peg-shaped incisor.

Patient M.M. was recruited by the Orthodontic Program of the University of Campania, Luigi Vanvitelli and gave written informed consent for the present study and for her to publish a photograph of her smile. MM showed the investigated clinical conditions (missing one upper lateral incisor and contralateral peg-shaped), also, she was the most recently treated patient with these characteristics. The intraoral digital models of both arches of the patient were taken using a 3Shape scanner (TRIOS 3 Basic). The STL files were transferred to BluSkyPlan4 software (v. 4.7.55, Wellington, New Zealand) to create a digital setup of five different clinical conditions. A frontal photograph of the patient with the smile was obtained with a Nikon D750 and a 105-mm lens. The nose and chin were removed from the picture as confounding factors. The patient's original final smile showed a missing upper right lateral incisor and an upper left lateral peg-shaped incisor (Figure 1A).

**Figure 2:**
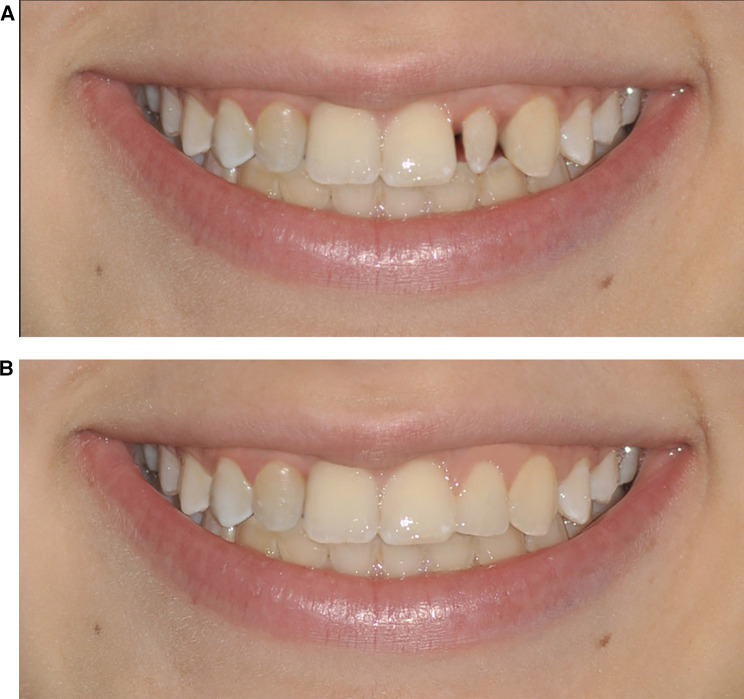
**(A)** Photographs n.1 and n.8. Space closure of the upper right lateral incisor with mesialization of the canine and maintenance of the upper left lateral peg-shaped incisor. **(B)** Photographs n. 2 and n. 4. Space closure of the upper right lateral incisor with the mesialization of the canine and the aesthetic rehabilitation of the upper left lateral peg-shaped incisor.

**Figure 3:**
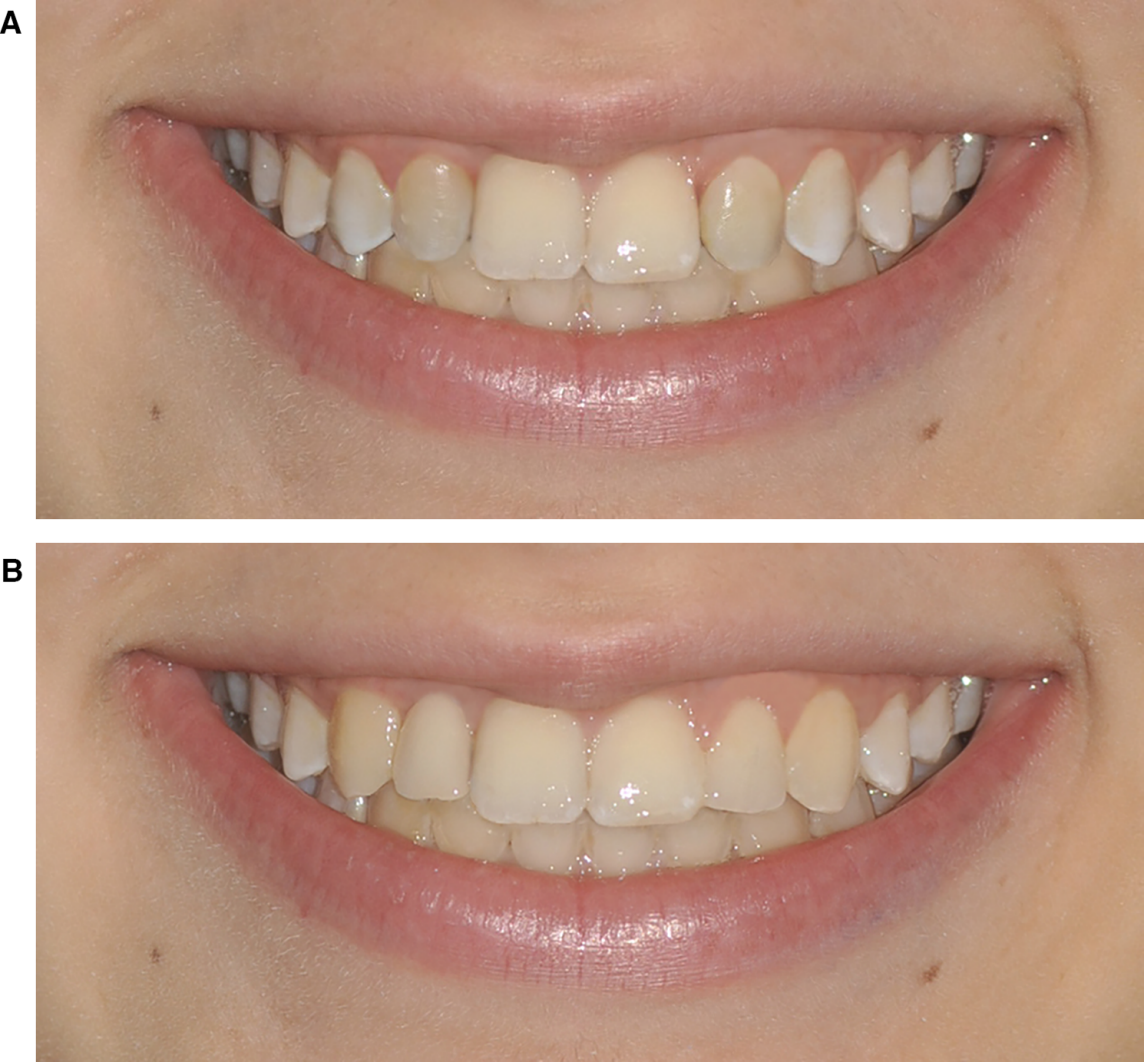
**(A)** Photographs n.6 and n.10. Space closure of the upper right lateral incisor with mesialization of the canine and extraction of the upper left lateral peg-shaped incisor with mesialization of the canine. **(B)** Photographs n.11 and n.12. Space opening of the upper right lateral incisor with implant rehabilitation and aesthetic rehabilitation of the upper left lateral peg-shaped incisor.

Image-editing software (Adobe Photoshop CC2018, Adobe System, San Josè, CA, USA) was used to generate the morphological alterations and reproduce the different clinical situations created with the following digital setup:

• Simulation 1: Space closure of the upper right lateral incisor with mesialization of the canine and maintenance of the upper left lateral peg-shaped incisor (Figure 2A).
• Simulation 2: Space closure of the upper right lateral incisor with mesialization of the canine and aesthetic rehabilitation of the upper left lateral peg-shaped incisor (Figure 2B).
• Simulation 3: Space opening of the upper right lateral incisor with implant rehabilitation and maintenance of the upper left lateral peg-shaped incisor (Figure 1B).
• Simulation 4: Space closure of the upper right lateral incisor with mesialization of the canine and extraction of the upper left lateral peg-shaped incisor with mesialization of the canine (Figure 3A).
• Simulation 5: Space opening of the upper right lateral incisor with implant rehabilitation and aesthetic rehabilitation of the upper left lateral peg-shaped incisor (Figure 3B).

The photograph modifications were made without respecting the anterior golden proportions. According to the literature, the proportions of the population with a smile aesthetic were 36%–40% in patients without agenesis and 18%–14% in patients with agenesis (9). The authors did not respect the anterior golden proportion to create a more realistic aesthetic representation of the different smiles. In space closure simulations, the canine was digitally bleached, and the cusp was rounded.

The authors preferred not to simulate the cuspid restoration to resemble a lateral incisor, because the quality of the restoration depends on the clinician's skills, which could introduce a bias in the aesthetics evaluation. Also, not all the patients want to restore it.

Each photograph was duplicated (photographs n. 1 and n. 8, n. 2 and n. 4, n. 3 and n. 9, n. 5 and n. 7, n. 6 and n. 10, n. 11 and n. 12) to evaluate each of 2 bipolar adjectives: unattractive-attractive and unpleasant-pleasant. Each photograph was evaluated with a visual analog scale with a minimum value of 1 and a maximum value of 10. The twelve total photographs were used to create a survey using online software (surveylegend.com). The photographs were randomly distributed through an online software (https://randraw.it). The survey was carried out completely online using a dedicated link. The link of the survey was spread by e-mail, messages and through social media (Facebook, Instagram). Participants to the survey were anonymous and they were divided into three different categories: orthodontic specialists, general dentists and laypeople. Once the survey started, there was no possibility to return to the previous photos to change their evaluation, and there were no time limits to finish it. The conditions for completing the survey were explained at the beginning. Only fully completed questionnaires were considered. Questionnaires presenting photographs without evaluation were excluded. At the end of the survey, the contributors provided their informed consent to analyze and publish the survey results (Supplementary Figure S1).

### Preliminary analysis and sample size calculation

The survey was preliminarily submitted to 11 orthodontics specialists, 11 general dentists and 11 laypeople to generate a more reliable calculation of the sample size. All the responders completed the entire survey. The analysis of this small sample showed significant differences among the three subgroups through analysis of variance (ANOVA) for each image. The sample size was calculated for each photo, considering the mean score differences among the groups. A maximum sample size of 327 subjects (109 in each group) was obtained, which might be helpful for detecting significant differences in the mean score among the three groups (orthodontic specialists, general dentists and laypeople) with a power [1-β(0.20)] of 80% and a level of significance of 0.05 (*α*).

### Statistical analysis

Categorical data are presented as numbers and percentages; continuous data are shown as the mean ± standard deviation (SD). The sample was divided into three subgroups (orthodontic specialists, general dentists and laypeople). A chi-square test was used to analyze the differences in categorical data. One-way ANOVA was used to analyze the differences in mean scores for each image in the three subgroups. Welch's correction was applied. To evaluate the differences within the three subgroups, a *post hoc* analysis with Bonferroni correction of the ANOVA test was used. Mean paired *t*-test was used in order to assess the mean differences among treatment options in each group. Pearson's correlation coefficient was calculated to evaluate the correlation of scores given for each photograph to establish the concordance of scoring of the participants in evaluating the duplicated photographs submitted in the survey. All analyses were performed using SPSS 20.0 for Windows (SPSS Inc., Chicago, IL). A 2-sided *P* value < 0.05 was considered to indicate statistical significance.

## Results

The survey was compiled by 386 participants—118 orthodontic specialists, 116 general dentists and 152 laypeople. All participants who did not complete the survey correctly (i.e., all the photos had the same evaluation) were excluded. Finally, the statistical analysis was conducted on 359 participants, including 109 orthodontic specialists, 109 general dentists and 141 laypeople. A total of 2.5% of the subjects were younger than 20 years, 21% were aged between 20 and 30 years, 43.7% were aged between 30 and 40 years, 9.7% were aged between 40 and 50 years, and 23.1% were aged older than 50 years. (Supplementary Figure S2)

Among the photographs submitted to the participants, only photographs n. 2 and n. 4 (Figure 2B, as unilateral canine mesialization and aesthetic rehabilitation of the peg-shaped lateral incisor), n. 6 and n. 10 (Figure 3A, as extraction of the peg-shaped lateral incisor and bilateral canine mesialization), and n. 11 and n. 12 (Figure 3B, as implant-prosthetic rehabilitation of the missing lateral incisor and aesthetic rehabilitation of the peg-shaped lateral incisor) reached statistical significance in terms of the mean differences in the scores of the three groups. In photographs n. 2 and n. 4, the laypeople and the orthodontic specialists gave a significantly greater score to the photo with respect to the general dentists. In photographs n. 6 and n. 10 laypeople gave a significantly greater score than orthodontic specialists and general dentists. Photographs n. 11 and n. 12 confirmed the higher scores given by laypeople and orthodontic specialists with respect to general dentists. No significant differences among the three subgroups were found in the remaining submitted photographs (Table 1).

**Table 1:** ANOVA test for each photograph scoring in the three subgroups (orthodontic specialists, general dentists, laypeople).

Photographs	All subjects (n. 359)	Orthodontic Specialists (n.109)	General Dentists (n.109)	Laypeople (n.141)	*p*-value*^α^*	*p*-value*^β^*
n. 1	2.49 ± 1.75	2.28 ± 1.77	2.44 ± 1.58	2.70 ± 1.85	0.172	0.195
n. 2	5.43 ± 2.18	5.41 ± 2.28	4.86 ± 1.94**	5.89 ± 2.19	**0**.**001**	**0**.**001**
n. 3	1.62 ± 1.17	1.66 ± 1.31	1.51 ± 1.08	1.67 ± 1.12	0.533	0.500
n. 4	5.46 ± 2.25	5.32 ± 2.36	4.90 ± 1.97**	5.99 ± 2.28^#^	**<0**.**001**	**<0**.**001**
n. 5	2.54 ± 1.71	2.46 ± 1.83	2.50 ± 1.56	2.60 ± 1.73	0.812	0.826
n. 6	5.19 ± 2.24	5.10 ± 2.18*	4.39 ± 1.95**	5.88 ± 2.29^###^	**<0**.**001**	**<0**.**001**
n. 7	2.57 ± 1.72	2.55 ± 1.82	2.61 ± 1.59	2.56 ± 1.76	0.967	0.965
n. 8	2.62 ± 1.75	2.54 ± 1.94	2.62 ± 1.54	2.68 ± 1.77	0.826	0.845
n. 9	1.82 ± 1.43	2.01 ± 1.78	1.80 ± 1.35	1.69 ± 1.13	0.216	0.269
n. 10	5.36 ± 2.27	5.02 ± 2.28	4.61 ± 2.07**	6.22 ± 2.12^##^	**<0**.**001**	**<0**.**001**
n. 11	6.84 ± 1.98	6.87 ± 1.73***	6.41 ± 2.15^###^	7.14 ± 1.99	**0**.**016**	**0**.**025**
n. 12	6.96 ± 1.90	6.76 ± 1.84	6.65 ± 1.97^###^	7.36 ± 1.84***	**0**.**007**	**0**.**007**

The bold values mean statistically significant results.

^α^ ANOVA test significance.

^β^ WELCH correction of ANOVA test significance.

* Bonferroni correction significance between orthodontic specialist and general dentist: *p* = 0.04.

** Bonferroni correction significance between general dentist and laypeople: *p* ≤ 0.001.

*** Bonferroni correction significance between orthodontic specialist and laypeople: *p* = 0.05.

^#^ Bonferroni correction significance between orthodontic specialist and laypeople: *p* = 0.05.

^##^ Bonferroni correction significance between orthodontic specialist and laypeople: ≤ 0.001.

^###^ Bonferroni correction significance between general dentist and laypeople: *p* ≤ 0.01.

The scores of duplicate photos were added together to evaluate the overall opinions about the six different photographs. The unilateral canine mesialization (sum of photos n. 2 and n. 4), the bilateral canine mesialization (sum of photos n. 6 and n. 10) and the space opening with implant-prosthetic rehabilitation (sum of photos n. 11 and n. 12) reached statistical significance among groups (Table 2).

**Table 2:** Sum of scores of duplicated photographs and relative ANOVA test.

Duplicates’ scoring sum	All subjects (n. 359)	Orthodontic specialists (n.109)	General dentists (n.109)	Laypeople (n.141)	*p*-value^α^	*p*-value^β^
n. 1 and n. 8	5.11 ± 3.28	4.83 ± 3.46	5.06 ± 2.88	5.36 ± 3.43	0.452	0.486
n. 2 and n. 4	10.89 ± 4.21	10.73 ± 4.27	9.76 ± 3.73**	11.88 ± 4.30	**<0**.**001**	**<0**.**001**
n. 3 and n. 9	3.44 ± 2.40	3.68 ± 2.84	3.31 ± 2.24	3.36 ± 2.14	0.476	0.546
n. 5 and n. 7	5.11 ± 3.31	5.01 ± 3.53	5.15 ± 3.00	5.16 ± 3.37	0.928	0.933
n. 6 and n. 10	10.54 ± 4.28	10.11 ± 4.19*	8.98 ± 3.75**	12.10 ± 4.24	**<0**.**001**	**<0**.**001**
n. 11 and n. 12	13.78 ± 3.72	13.61 ± 3.39	13.01 ± 3.97***	14.50 ± 3.62	**0**.**007**	**0**.**01**

The bold values mean statistically significant results.

^α^ ANOVA test significance.

^β^ WELCH correction of ANOVA test significance.

* Bonferroni correction significance between orthodontic specialist and laypeople: *p* ≤ 0.001.

** Bonferroni correction significance between general dentists and laypeople: *p* ≤ 0.001.

*** Bonferroni correction significance between general dentist and laypeople: *p* = 0.006.

**Figure 4:**
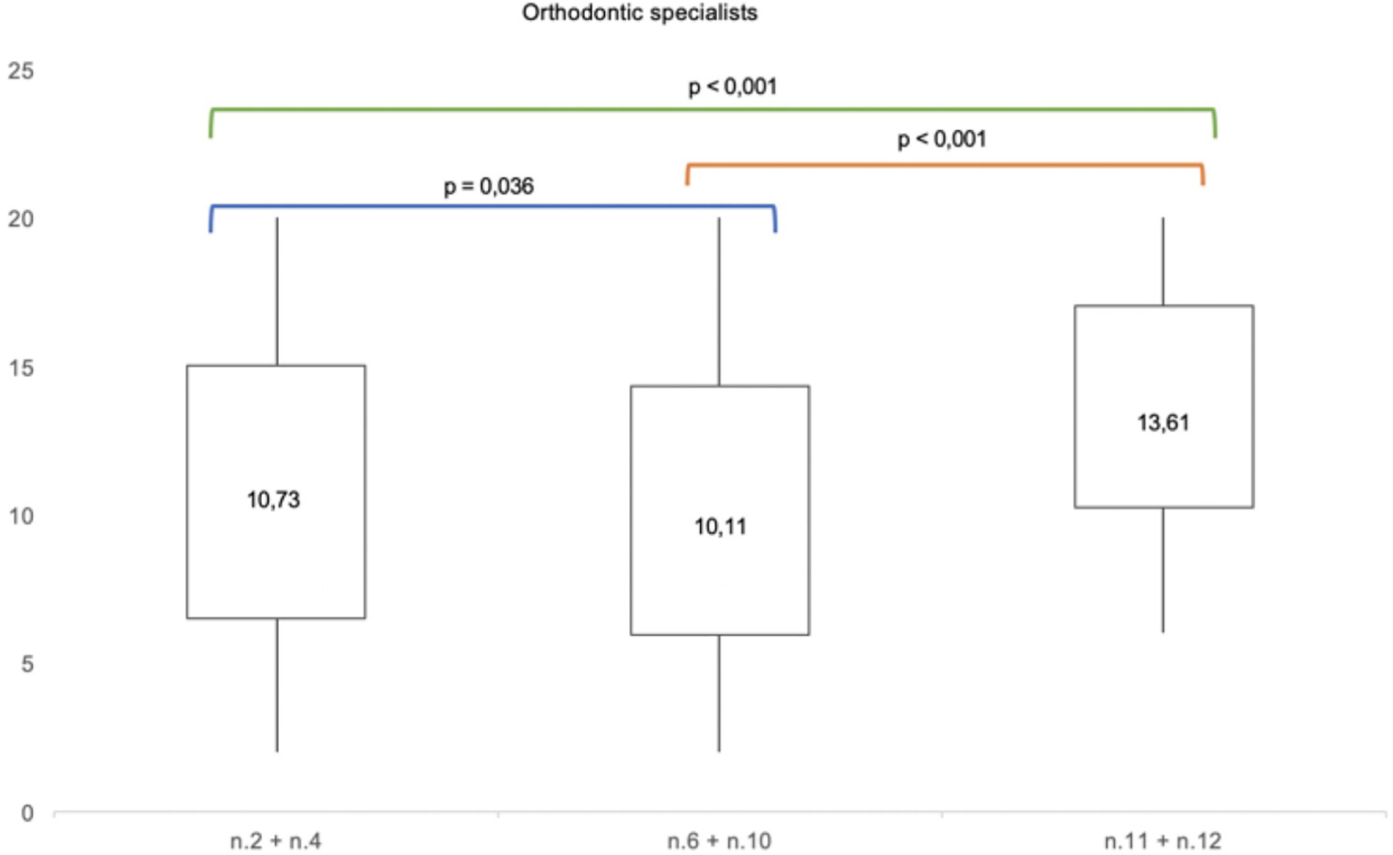
Mean paired *t*-test comparing the mean differences of aesthetic perception rates among the photographs n.2 + n.4, n.6 + n.10 and n. 11 + n.12 in orthodontic specialists.

**Figure 5:**
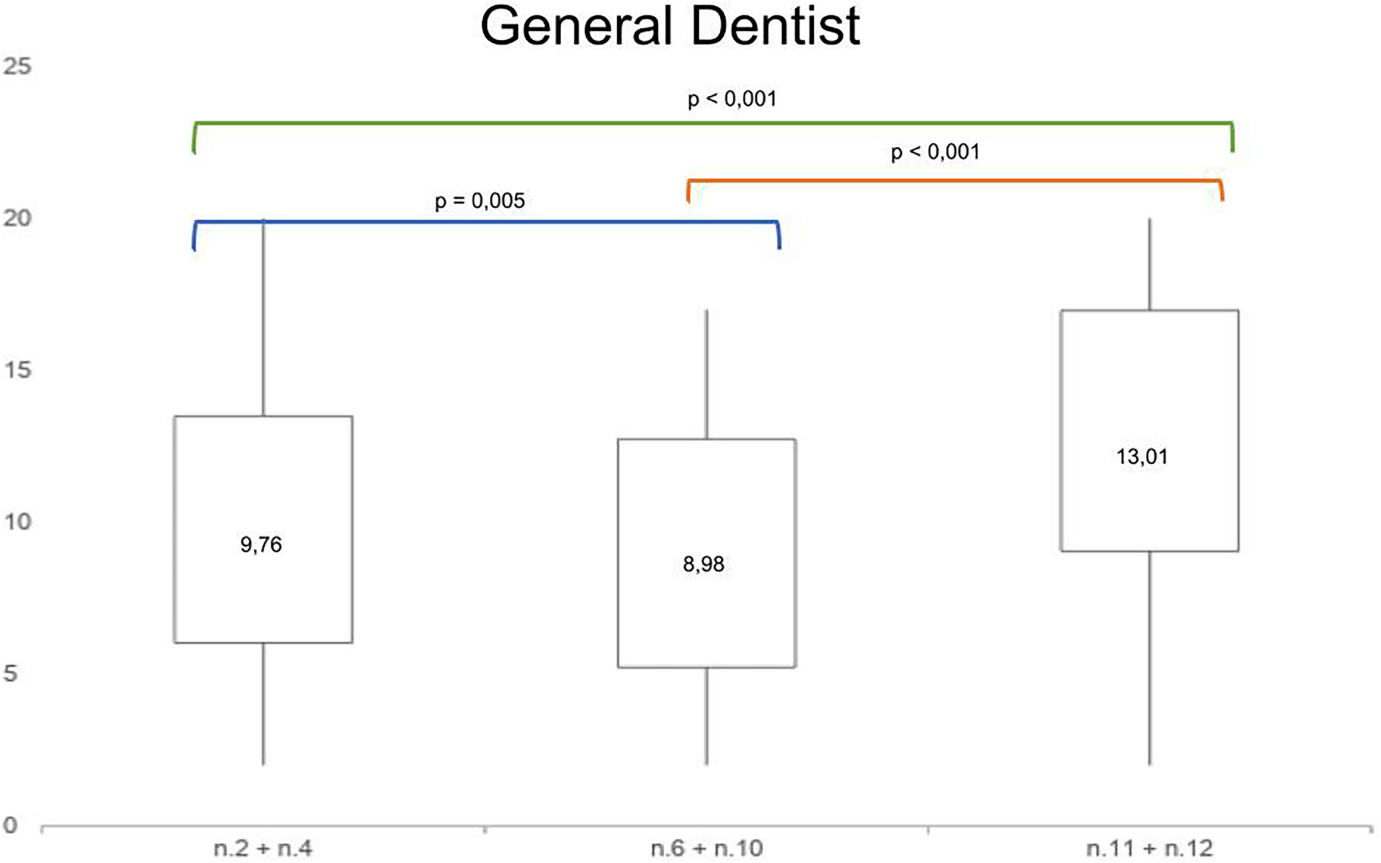
Mean paired *t*-test comparing the mean differences of aesthetic perception rates among the photos n.2 + n.4, n.6 + n.10 and n.11 + n.12 in general dentists.

Moreover, in orthodontic specialists and in general dentists groups the analysis of mean paired differences among the scores of the treatment options (unilateral canine mesialization, bilateral canine mesialization and space opening with implant-prosthetic rehabilitation) showed statistical significance, as shown in Figures 4, 5.

**Figure 6:**
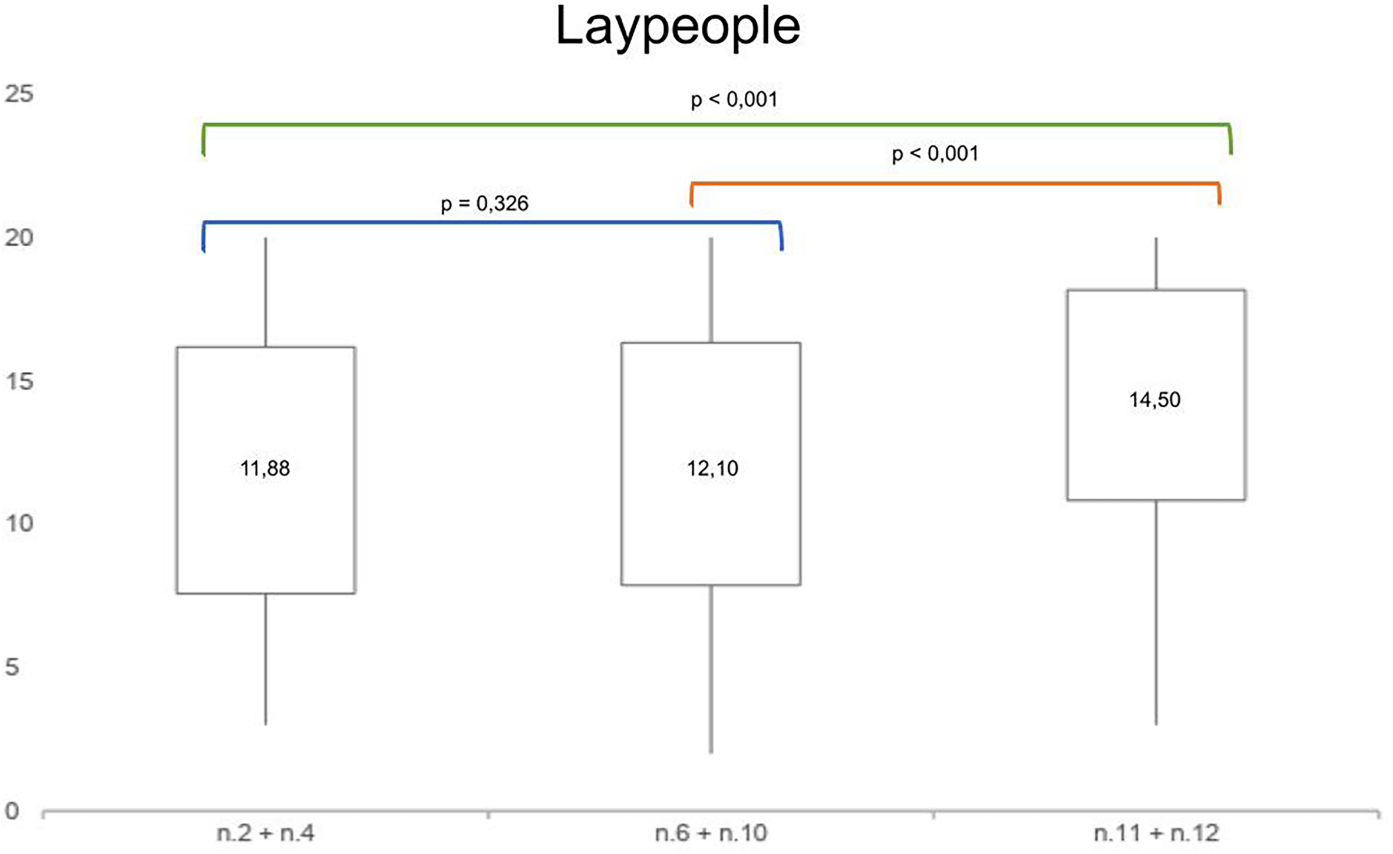
Mean paired *t*-test comparing the mean differences of aesthetic perception rates among the photos n. 2 + n. 4, n. 6 + n. 10 and n. 11 + n. 12 in laypeople.

In the group of laypeople, the analysis of mean paired differences among the scores of unilateral and bilateral canine mesialization didn't show a statistical significance. Conversely, the analysis of mean paired differences among the scores of the other treatment options reached a statistical significance (Figure 6).

Dividing our population according to age groups (< 30 years old, 30–40 years old and > 40 years old), the scores of the unilateral canine mesialization and the scores of bilateral canine mesialization confirmed statistically significant differences in scores within orthodontic specialist, general dentist and laypeople subgroups. The scores of the space opening with implant-prosthetic rehabilitation reached statistical significance only in the > 40 years old group. (Supplementary Tables S1–S3)

Finally, we analyzed the correlation among the scores of the duplicate photographs submitted to the survey, and all the correlations were statistically significant. The mean correlation coefficient of each group showed that laypeople had the highest correlation coefficient (R = 0.828 ± 0.032), but there were no statistically significant differences within the groups (*p* = 0.10) (Table 3).

**Table 3:** Pearson's correlation coefficient between the duplicated photographs in all patients and in the three subgroups.

Duplicates’ comparisons	All subjects (n. 359)	*p*-value	Orthodontic specialists (n.109)	*p*-value	General dentists (n.109)	*p*-value	Laypeople (n.141)	*p*-value
n. 1 and n. 8	R = 0.754	**<0** **.** **001**	R = 0.735	**<0**.**001**	R = 0.717	**<0**.**001**	R = 0.794	**<0**.**001**
n. 2 and n. 4	R = 0.801	**<0**.**001**	R = 0.697	**<0**.**001**	R = 0.825	**<0**.**001**	R = 0.856	**<0**.**001**
n. 3 and n. 9	R = 0.707	**<0**.**001**	R = 0.676	**<0**.**001**	R = 0.694	**<0**.**001**	R = 0.793	**<0**.**001**
n. 5 and n. 7	R = 0.858	**<0**.**001**	R = 0.871	**<0**.**001**	R = 0.817	**<0**.**001**	R = 0.874	**<0**.**001**
n. 6 and n. 10	R = 0.796	**<0**.**001**	R = 0.758	**<0**.**001**	R = 0.739	**<0**.**001**	R = 0.828	**<0**.**001**
n. 11 and n. 12	R = 0.836	**<0**.**001**	R = 0.807	**<0**.**001**	R = 0.869	**<0**.**001**	R = 0.827	**<0**.**001**
Mean values (*p* = 0.10)			R = 0.757 ± 0.072		R = 0.776 ± 0.077		R = 0.828 ± 0.032	

R, Pearson's correlation coefficient.

The bold values mean statistically significant results.

## Discussion

The missing maxillary lateral incisor is a common clinical challenge for orthodontists, offering various treatment options. This condition is often associated with a contralateral microdontic or peg-shaped incisor, as well as a slight generalized reduction in dental diameter (6, 7, 32). Although several studies have explored the aesthetic impact and the treatment choices of congenital maxillary lateral incisor agenesis, only few case reports described the aesthetic perception of smile treatments involving agenesis with a contralateral peg-shaped incisor (2, 3, 31, 33, 34). Such cases complicate treatment options, requiring clinicians to balance both technical and aesthetic considerations, always in line with the patient's skeletal and dental assessment (35, 36).

The therapeutic options evaluated in our survey represent the primary approaches that clinicians should consider during the initial decision-making process, including the following:

1. Maintenance of the agenesis space with subsequent implant-prosthetic or prosthetic rehabilitation and conservative aesthetic reshaping of the contralateral incisor.
2. Closure of the space of the agenesis with mesialization of the middle and posterior sectors and aesthetic conservative reshaping of the contralateral incisor.
3. Closure of the space of the agenesis and extraction of the contralateral incisor with mesialization of the middle and posterior sectors of both sides.

Two of the treatment options achieved a symmetrical smile aesthetics, whereas the third resulted in an asymmetrical smile aesthetic at the end of the treatment

The results of our survey, submitted to orthodontic specialists, general dentists and laypeople, highlighted that the most appreciated smile was the one with the missing incisor replaced by prosthetic rehabilitation and the contralateral reshaped with an aesthetic conservative restoration. This treatment solution reproduced the typical ‘high-low-high’ gingival contour and the normal symmetrical array of the anterior teeth, and it was expected that it would be the most appreciated, even if the missing right lateral incisor was replaced by a prosthetic rehabilitation (34, 37).

Despite this solution provides the best smile aesthetics, it is not free from long-term issues related to implant-prosthetic rehabilitation and it should be strictly considered by the clinician. Implants in the aesthetic zone is an operator-sensitive procedure that can be associated with several complications or even failure due to various factors: continued craniofacial growth, particularly in the maxilla, leading to the apparent submersion of the implant crown as natural teeth move incisally, in an unpredictable ways; peri-implantitis, with a patient-level prevalence of nearly 25% according to a recent systematic review (38); thinning and recession of peri-implant mucosa due to poor placement, prosthetic management, or case selection, often affecting aesthetics and increasing the risk of peri-implant diseases (2, 28); and mechanical failure of the implant components. Additionally, once an implant is placed in the anterior maxilla, it precludes palatal expansion in adult patients, as the space created cannot be redistributed orthodontically (39). The real challenge was the comparison of the other two treatment options (unilateral canine mesialization and bilateral canine mesialization), considering that all the studies reported in the literature debated the aesthetic perception about the symmetry of the smile (13, 40). The smile with monolateral canine mesialization (Figure 2B) was preferred by both the orthodontic specialists and general dentists, while the smile with bilateral canine mesialization (Figure 3A) was the favorite of the laypeople. Most likely, the specialists in orthodontics and the general dentists assigned greater value to the monolateral mesialization of the canine because there was better preservation of the aesthetic architecture of the smile, even if it was asymmetrical. However, laypeople seemed to prefer a symmetrical aesthetic of the smile, even if both lateral incisors replaced by the canines altered the typical aesthetic structure of the smile, which has been confirmed by several studies (13, 40–43). Machado et al. confirmed that laypeople were rigorous in identifying asymmetries, even when the alterations were minimal (37).

The group of orthodontic specialists gave lower evaluations in general than did the other two groups. This aligns with the findings of Kokich and Ribeiro, who demonstrated that orthodontists are more precise in identifying aesthetic alterations of the smile, generally assigning lower scores (14, 44). Another critical factor in evaluating smile attractiveness is the morphological characteristics of the canines (45). Brough et al. showed that narrowed and brighter canines were deemed the most attractive by all groups (25). Rayner et al. suggested that when canines were modified to simulate a lateral incisor, the smile aesthetic was well judged by both professionals and laypeople. However, laypeople tended to be less critical of dental appearance, as corroborated by our survey (8).

In this context, a limitation of our survey was that the morphology of the canines was not extensively modified when the lateral incisor was replaced (apart from digital bleaching and rounding of the cusps), which may have introduced bias into the evaluations. In contrast, it was necessary to consider that not all patients with a canine in substitution for a lateral incisor agreed to modify the canine morphology. Furthermore, the quality of the restoration depends heavily on the clinician's skill, which could also influence the evaluation of aesthetics perception.

The aesthetic perception of treatment options across different age subgroups (< 30 years, 30–40 years, and > 40 years) supported the observed trend. Orthodontic specialists and general dentists in all three subgroups rated unilateral canine mesialization more favorably, whereas laypeople preferred bilateral canine mesialization. The < 30 years group consistently assigned higher ratings to all the evaluated photographs. Overall, our findings indicate that aesthetic preferences did not vary significantly across the different age groups. These findings were corroborated by the study of Sriphadungporn and Chamnannidiadha that found no statistical difference in the participants’ preference for vertical incisal edge position. Age did not affect the perception of smiles when varying this variable and both groups shared similar preferences (46).

It should be noted that laypeople demonstrated a better correlation coefficient in the two evaluations of the duplicated photograph than did the orthodontic specialists and the general dentists, as reported in Table 2. These results revealed that laypeople were more focused on specific details of the smile, while the two other groups provided a general evaluation of the aesthetics of the smile, with a worse correlation of the duplicated photos (47). Another possible explanation for these findings could be that orthodontic specialists and general dentists were routinely involved in smile evaluation in their clinical practice, and for this reason, they probably undervalued the survey.

## Conclusion

This study analyzed the aesthetic perception of treatment options for maxillary lateral incisor agenesis with contralateral peg-shaped incisors, involving orthodontists, general dentists, and laypeople. The main findings are summarized as follows:

1. Aesthetic Preferences:
  1. Implant-supported prosthetic rehabilitation with aesthetic reshaping of the contralateral incisor was rated as the most aesthetically pleasing option across all groups due to its preservation of symmetry and natural gingival contour, as expected.
  2. Unilateral canine mesialization was preferred by orthodontists and general dentists, likely due to better preservation of smile architecture despite asymmetry.
  3. Bilateral canine mesialization was favored by laypeople, who prioritized symmetry over adherence to typical dental aesthetics.
2. Differences Among Groups:
  ○ Orthodontists assigned the lowest scores overall, reflecting greater sensitivity to subtle aesthetic alterations.
  ○ Laypeople demonstrated higher consistency in evaluating duplicate photographs, likely due to a focus on specific smile details, whereas experts provided broader assessments.
3. Age Influence:
  ○ Aesthetic preferences showed no significant differences across age groups. Although participants under 30 consistently provided higher ratings for all treatments with respect to the older groups.
4. Limitations and Clinical Implications:
  ○ The limited modification of canine morphology may have influenced aesthetic evaluations. Although a bias in the aesthetics evaluation could be introduced by the quality of the restoration (depending on the clinician's skills)
  ○ Implant-supported prosthetic solutions, while considered by all groups as aesthetically optimal, can pose long-term risks such as peri-implant complications, mechanical failures…
5. Recommendations for Clinical Practice:
  ○ Treatment selection should balance aesthetic outcomes, long-term predictability, and patient preferences.
  ○ Clear communication with patients about the aesthetic and functional implications of each option is essential, especially as laypeople preferences may differ from clinical evaluations.

This study highlights the need for a personalized, multidisciplinary approach to the management of maxillary lateral incisor agenesis, considering both technical and aesthetic factors alongside patient expectations.

## Data Availability

The original contributions presented in the study are included in the article/Supplementary Material, further inquiries can be directed to the corresponding author.

## Supplementary material

Supplementary Figure S1 Materials and methods workflow.

Supplementary Figure S2 Survey participants (n) and demographic characteristics of our population (%).

## References

[1] Beltrami F Antonarakis GS Kiliaridis S. Prevalence, distribution, and age at clinical detection of missing permanent incisors. Eur J Orthod. (2021) 43(1):25–8. 10.1093/ejo/cjaa006 32006441

[2] Migliorati M Zuffanti A Capuano M Canullo L Caponio VCA Menini M. Periodontal, occlusal, and aesthetic outcomes of missing maxillary lateral incisor replacement: a systematic review and network meta-analysis. Int Orthod. (2024) 23(1):100957. 10.1016/j.ortho.2024.100957 39667155

[3] Thierens LAM Verhoeven B Temmerman L De Pauw GAM. An esthetic evaluation of unilateral canine substitution for a missing maxillary lateral incisor. J Esthet Restor Dent. (2017) 29(6):442–9. 10.1111/jerd.12324 28858425

[4] Polder BJ Van't Hof MA Van Der Linden FPGM. Kuijpers-Jagtman AMA meta-analysis of the prevalence of dental agenesis of permanent teeth. Community Dent Oral Epidemiol. (2004) 32(3):217–26. 10.1111/j.1600-0528.2004.00158.x 15151692

[5] Fekonja A. Comparison of mesiodistal crown dimension and arch width in subjects with and without hypodontia. J Esthet Restor Dent. (2013) 25:203–10. 10.1111/jerd.12026 23773516

[6] Al-Ani AH Safwat Antoun J Murray Thomson W Raymond Merriman T Farella M Lay PY Hypodontia: an update on its etiology, classification, and clinical management. BioMed Res Int. (2017) (1):9378325. 10.1155/2017/9378325 28401166 PMC5376450

[7] Bozkaya E Bavbek NC Ulasan B. New perspective for evaluation of tooth widths in patients with missing or peg-shaped maxillary lateral incisors: quadrant analysis. Am J Orthod Dentofacial Orthop. (2018) 154(6):820–8. 10.1016/j.ajodo.2018.02.012 30477780

[8] Rayner WJ Barber SK Spencer RJ. The effect of canine characteristics and symmetry on perceived smile attractiveness when canine teeth are substituted for lateral incisor. J Orthod. (2015) 42:22–32. 10.1179/1465313314Y.0000000118 25808380

[9] Pini NP De Marchi LM Gribel BF Ubaldini ALM Pascotto RC. Analysis of the golden proportion and width/height ratios of maxillary anterior dentition in patients with lateral incisor agenesis. J Esthet Restor Dent. (2012) 24(6):402–14. 10.1111/j.1708-8240.2012.00533.x 23205688

[10] Göranson E Sonesson M Naimi-Akbar A Dimberg L. Malocclusions and quality of life among adolescents: a systematic review and meta-analysis. Eur J Orthod. (2023) 45(3):295–307. 10.1093/ejo/cjad009 36995692 PMC10230246

[11] Machado AW. 10 commandments of smile esthetics. Dental Press J Orthod. (2014) 19(4):136–57. 10.1590/2176-9451.19.4.136-157.sar 25279532 PMC4296640

[12] Ricketts RM. The biologic significance of the divine proportion and fibonacci series. Am J Orthod. (1982) 81(5):351–70. 10.1016/0002-9416(82)90073-2 6960724

[13] Magne P Salem P Magne M. Influence of symmetry and balance on visual perception of a female smile. J Prosthet Dent. (2018) 120(4):573–82. 10.1016/j.prosdent.2018.05.008 30314610

[14] Kokich VO Jr Kiyak HA Shapiro PA. Comparing the perception of dentists and lay people to altered dental esthetics. J Esthet Dent. (1999) 11:311–24. 10.1111/j.1708-8240.1999.tb00414.x 10825866

[15] Koseoglu M Bayindir F. Effects of gingival margin asymmetries on the smile esthetic perception of dental professionals and lay people. J Esthet Restor Dent. (2020) 32(5):480–6. 10.1111/jerd.12595 32596944

[16] Nomura S Freitas KMS da Silva PPC Valarelli FP Cançado RH de Freitas MR Evaluation of the attractiveness of different gingival zeniths in smile esthetics. Dental Press J Orthod. (2018) 23(5):47–57. 10.1590/2177-6709.23.5.047-057.oar 30427493 PMC6266316

[17] Zachrisson BU Rosa M Toreskog S. Congenitally missing maxillary lateral incisor: canine substitution. Am J Orthod Dentofacial Orthop. (2011) 139:438. 10.1016/j.ajodo.2011.02.003 21457853

[18] Rosa M Zachrisson BU. Integrating esthetic dentistry and space closure in patients with missing maxillary lateral incisor. J Clin Orthod. (2001) 35(4):221–34. 11345569

[19] Rosa M Zachrisson BU. Integrating esthetic dentistry and space closure in patients with missing maxillary lateral incisor: further improvements. J Clin Orthod. (2007) 41:563–73. 17921603

[20] Kokich VO Jr Kinzer GA Janakievskic J. Congenitally missing maxillary lateral incisors: restorative replacement. Am J Orthod Dentofac Orthop. (2011) 139(4):435–45. 10.1016/j.ajodo.2011.02.004 21457854

[21] Robertsson S Mohlin B. The congenitally missing upper lateral incisor. A retrospective study of orthodontic space closure versus restorative treatment. Eur J Orthod. (2000) 22(6):697–710. 10.1093/ejo/22.6.697 11212605

[22] Priest G. The treatment dilemma of missing maxillary lateral incisors-part I: canine substitution and resin-bonded fixed dental prostheses. J Esthet Restor Dent. (2019) 31:311–8. 10.1111/jerd.12484 31033185

[23] Priest G. The treatment dilemma of missing maxillary lateral incisors-part II: implant restoration. J Esthet Restor Dent. (2019) 31:319–26. 10.1111/jerd.12483 31033174

[24] Ulhaq A Fee P Cresta M Turner S Dutta A. Dental factors influencing treatment choice for maxillary lateral incisor agenesis: a retrospective study. Eur J Prosthod Restor Dent. (2019) 27:182–8. 10.1922/EJPRD_01792Ulhaq07 31622054

[25] Brough E Donaldson AN Naini FB. Canine substitution for missing maxillary lateral incisor: the influence of canine morphology, size, and shade on perceptions of smile attractiveness. Am J Orthod Dentofac Orthop. (2010) 138(6):705e1–e9. 10.1016/j.ajodo.2010.04.027 21130320

[26] Andrade DCM Loureiro CA Araújo VE Riera R Atallah AN. Treatment for agenesis of maxillary lateral incisors: a systematic review. Orthod Craniofac Res. (2013) 16:129–36. 10.1111/ocr.12015 23406509

[27] Johal A Katsaros C Kuijpers-Jagtman AM, Angle Society of Europe membership. State of the science on controversial topics: missing maxillary lateral incisors – a report of the angle society of Europe 2012 meeting. Prog Orthod. (2013) 14:20. 10.1186/2196-1042-14-20 24325884 PMC4384898

[28] Grunder U Gracis S Capelli M. Influence of the 3-D bone-to-implant relationship on esthetics. Int J Periodontics Restor Dent. (2005) 25:113–9. 15839587

[29] Jamilian A Perillo L Rosa M. Missing upper incisor: a retrospective study of orthodontic space closure versus implant. Prog Orthod. (2015) 16:2. 10.1186/s40510-015-0072-2 25769117 PMC4385022

[30] Smidt A Najjar N Louzon Y. The esthetic challenge of a malpositioned peg-shaped lateral incisor and a missing contralateral maxillary tooth: report of a case. Quintessence Int. (2023) 54(5):394–9. 10.3290/j.qi.b3931381 36853625

[31] Sharab L Kutkut A Van Sickels J. Interdisciplinary management of an adult orthodontic patient: a case report and literature review. Gen Dent. (2023) 71(2):48–57. 36825974

[32] Mirabella AD Kokich VG Rosa M. Analysis of crown widths in subjects with congenitally missing maxillary lateral incisors. Eur J Orthod. (2012) 34:783–7. 10.1093/ejo/cjr094 21911843

[33] Qadri S Parkin NA Benson PE. Space closing versus space opening for bilateral missing upper laterals-aesthetic judgments of laypeople: a web-based survey. J Orthod. (2016) 43(2):137–46. 10.1080/14653125.2016.1145880 27380484

[34] Rosa M Olimpo A Fastuca R Caprioglio A. Perception of dental professionals and laypeople to altered dental esthetics in cases with congenitally missing maxilary lateral incisors. Prog in Orthod. (2013) 14:34. 10.1186/2196-1042-14-34 PMC4384961 24325825

[35] Silveira GS de Almeida NV Pereira DM Mattos CT Mucha JN Prosthetic replacement vs space closure for maxillary lateral incisor agenesis: a systematic review. Am J Orthod Dentofac Orthop. (2016) 150:228–37. 10.1016/j.ajodo.2016.01.018 27476355

[36] Ballanti F Lione R Fiaschetti V Fanucci E Cozza P. Low-dose CT protocol for orthodontic diagnosis. Eur J Paediatr Dent. (2008) 9(2):65–70. 18605887

[37] Machado AW Moon W Gandini LG Jr. Influence of maxillary incisor edge asymmetries on the perception of smile esthetic among orthodontists and laypersons. Am J Orthod Dentofac Orthop. (2013) 143:658–64. 10.1016/j.ajodo.2013.02.013 23631967

[38] Derks J Tomasi C. Peri-implant health and disease. A systematic review of current epidemiology. J Clin Periodontol. (2015) 42(supp| 16):s158–71. 10.1111/jcpe.12334 25495683

[39] Robbins JW. A case against the implant. Int J Periodontics Restorative Dent. (2024) 44(3):250–1. 10.11607/prd.2024.3.e 38787712

[40] Kokich VO Kokich VG Kiyak HA. Perceptions of dental professionals and laypersons to altered dental esthetic: asymmetric and symmetric situations. Am J Orthod Dentofac Orthop. (2006) 130:141–51. 10.1016/j.ajodo.2006.04.017 16905057

[41] Schneider U Moser L Fornasetti M Piattella M Siciliani G. Esthetic evaluation of implant vs canine substitution in patients with congenitally missing maxillary lateral incisors: are there any new insights? Am J Orthod Dentofac Orthop. (2016) 150(3):416–24. 10.1016/j.ajodo.2016.02.025 27585769

[42] Armbruster PC Gardiner DM Whitley JB Jr Flerra J. The congenitally missing maxillary lateral incisor. Part 1: esthetic judgment of treatment options. World J Orthod. (2005) 6:369–75. 16379208

[43] Armbruster PC Gardiner DM Whitley JB Jr Flerra J. The congenitally missing maxillary lateral incisor. Part 2: assessing dentists’ preferences for treatment. World J Orthod. (2005) 6:376–81. 16379209

[44] Ribeiro JB Figueiredo BA Machado AW. Does the presence of unilateral maxillary incisor edge asymmetries influence the perception of smile esthetic? J Esthet Restor Dent. (2017) 29(4):291–7. 10.1111/jerd.12305 28556552

[45] Sabouni W Hansa I Shah C Adel SM Vaiid N. Pink esthetic score evaluation of gingival esthetics after clear aligner treatment of palatally impacted canines. J Clin Orthod. (2024) 58(7):1000. 39191522

[46] Sriphadungporn C Chamnannidiadha N. Perception of smile esthetics by laypeople of different ages. Prog Orthod. (2017) 18:8. 10.1186/s40510-017-0162-4 28317085 PMC5357618

[47] Adel SM Bichu YM Pandian SM Sabouni W Shah C Vaiid N. Clinical audit of an artificial intelligence (AI) empowered smile simulation system: a prospective clinical trial. Sci Rep. (2024) 14(1):19385. 10.1038/s41598-024-69314-6 39169095 PMC11339289

