# Supplementary figures and images for: The aesthetic perception of orthodontic specialists, general dentists and laypeople regarding different smile displays for a patient missing one upper lateral incisor and the other one peg-shaped
Source: Front Dent Med. 2025 Mar 11;6:1532220. doi: 10.3389/fdmed.2025.1532220 (PMC11933056; doi:10.3389/fdmed.2025.1532220)

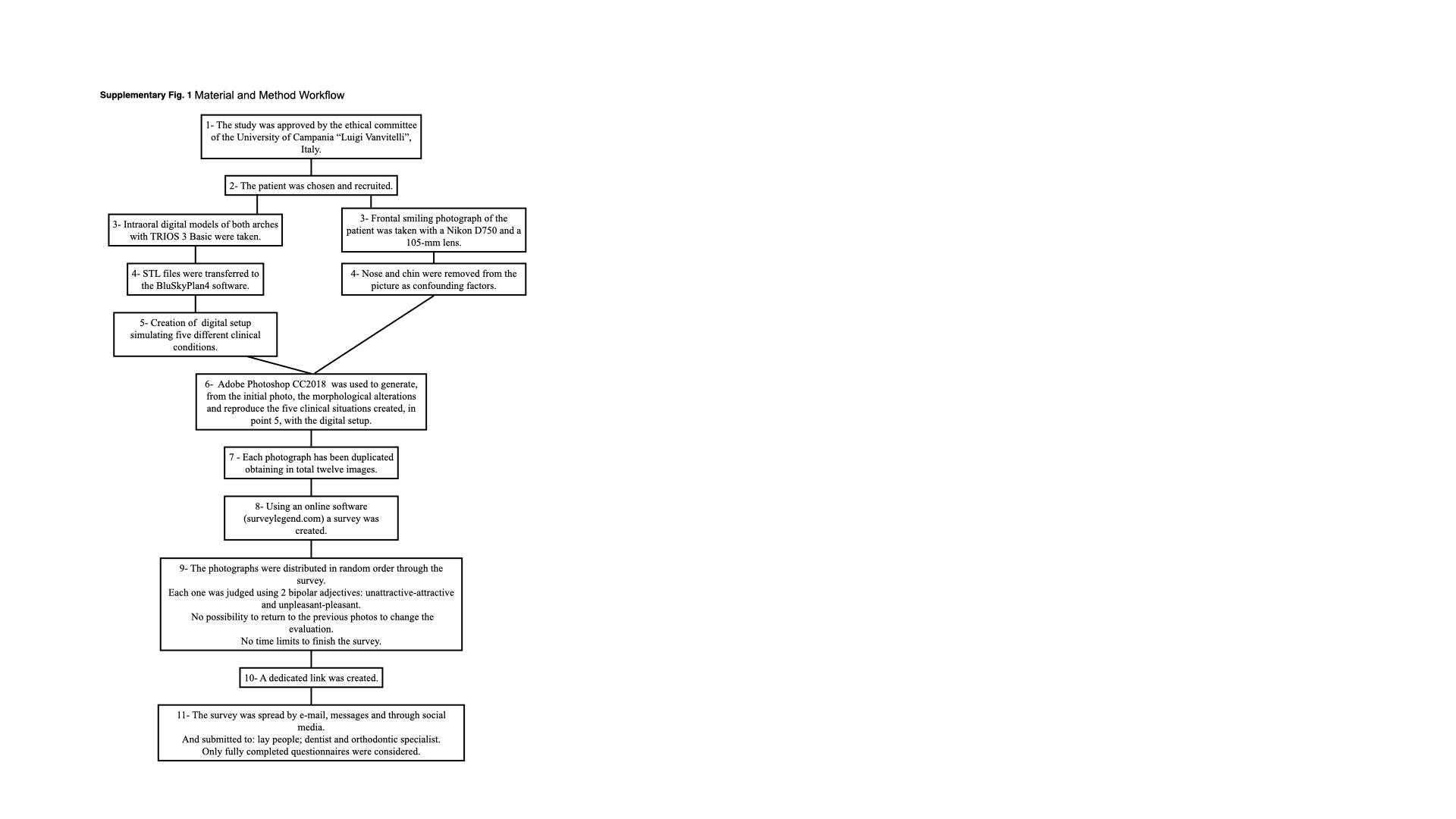

Supplement: Supplementary Figure S1 — Materials and methods workflow. [file Image1.png]

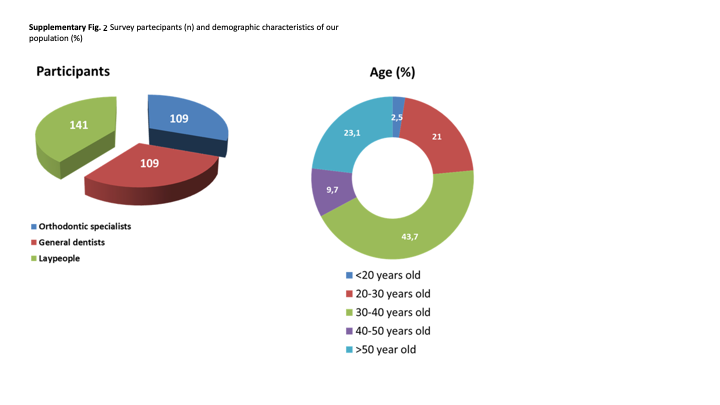

Supplement: Supplementary Figure S2 — Survey participants (n) and demographic characteristics of our population (%). [file Image2.png]

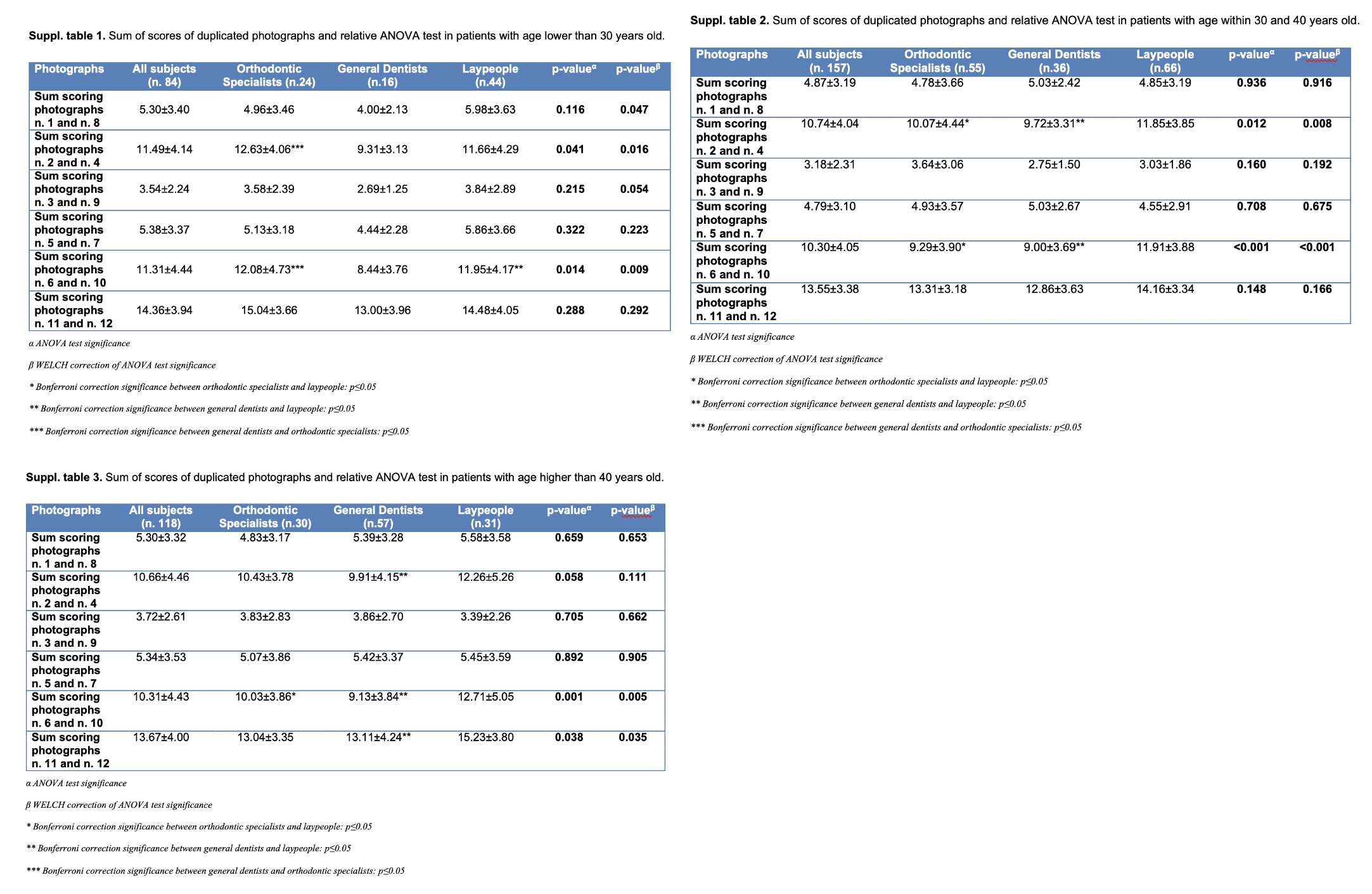

Supplement: Supplementary file 3 [file Image3.png]
